# Editorial: Factors affecting the efficacy of foliar fertilizers and the uptake of atmospheric aerosols, volume II

**DOI:** 10.3389/fpls.2023.1146853

**Published:** 2023-02-10

**Authors:** Yanbo Hu, Nacer Bellaloui, Yuanwen Kuang

**Affiliations:** ^1^ Northeast Forestry University, Harbin, China; ^2^ Crop Genetics Research Unit, Agricultural Research Service, U.S. Department of Agriculture (USDA), Stoneville, MS, United States; ^3^ South China Botanical Garden, Chinese Academy of Sciences (CAS), Guangzhou, China

**Keywords:** foliar-applied nutrients, leaf surface materials, chelating agents, hygroscopicity, atmospheric aerosols

Plants take up the macro- and micro-nutrients mainly by roots; however, restricted root uptake may commonly occur due to the limitation on nutrient availability. Considerable evidence supports the uptake and translocation of foliar-applied nutrients (Alexander and Schroeder, 1987; Oosterhuis, 2009; Fernández and Brown, 2013). Foliar fertilizer application, particularly in agriculture, is a routine measure to improve nutrient status of plants (Mengel, 2002). Foliar application of micro-nutrients is one of the most important strategies in biofortification techniques, because micro-nutrient requirements are better met by foliar application compared to macro-nutrient requirements (Mengel, 2002). However, there are still limitations, for instances, only 30, 20 and 35% utilization efficiency for nitrogen (N), phosphorus (P) and potassium (K), respectively (Azam et al., 2022), for foliar-applied inorganic fertilizers in uptake and translocation (Li et al., 2022). Improving foliar uptake and nutrient use efficiency is one of the greatest challenges in crop production systems nowadays (Al-Juthery et al., 2021). Understanding the factors affecting the uptake and efficacy of foliar-applied nutrients has the significance to improve plant nutrient status when plants are subjected to inadequate soil nutrient, biotic and/or abiotic stresses. Aerial plant deposition of the atmospheric aerosols is a common phenomenon for plants grown in urban or suburban areas. Exposure to atmospheric aerosols has diverse impacts on plants, involving the germination, growth, metabolism, physiology, and productivity (Hu et al., 2014; Burkhardt and Grantz, 2016; Chi et al., 2022). However, the metabolic networks and regulatory processes are complex. To date, the factors affecting the uptake and translocation of atmospheric aerosols are poorly understood. Therefore, we organized the Research Topic series, focused on the factors affecting the uptake and use efficiency of foliar fertilizers or atmospheric aerosols, and highlighted the underlying mechanisms (i.e., the types and models of foliar-applied nutrients and their uptake, movement, and metabolic pathways in leaves, and physiological function and productivity).

Macro-nutrients such as N and K are high-mobility and rapid-distribution into plant tissues after foliar application (Mengel, 2002). For N compounds such as urea, foliar-applied N solutions are highly permeable (Fernández and Brown, 2013); for instance, 30% foliar-applied ^15^N urea can be absorbed by leaves within one hour and rapidly transported to other plant tissues of cotton within 6−48 hours after application (Oosterhuis, 2009). Boron (B) compounds can be absorbed by crop leaves at a high rate, but with the limited mobility in some plant species (Fernández and Brown, 2013); for some species, foliar-applied B can be rapidly absorbed and then transported to sink tissues due to the high-mobility of phloem B (Fernández et al., 2013). In this Research Topic, Dhaliwal et al. evaluated the effects of foliar application of B and N mixtures on growth performance and nutritional quality of Indian mustard (*Brassica juncea* L.). They proposed an optimal model of foliar application (1.0% borax + 1.0% urea) at the flowering phase, showing the superiority in food quality (i.e., crude fiber, soluble solids, and proteins), seed oil quality and yield, and nutrient use efficiency. Foliar B application led to B accumulation in leaves and seed of soybean, directly or indirectly affected N and C metabolism, and further altered seed composition in proteins, fatty acids, and sugars (Bellaloui et al., 2013). In the study of Bellaloui et al. (2013), a high fraction of cell wall B to the total B occurred under both well-watered and water-stressed conditions, indicating the structural role of B. Calcium (Ca), sulfur (S), zinc (Zn), and iron (Fe) can be absorbed by leaves but showing a low mobility in the plants (Mengel, 2002).

In recent years, the chelating agents have been frequently used in agricultural production in order to enhance nutrient use efficiency and adaptation of crops (Zheng et al., 2016; Souri and Hatamian, 2019). In this Research Topic, Li et al. compared the effects of different foliar Ca applications (sorbitol-chelated Ca, calcium nitrate, and a mixture of sorbitol and calcium nitrate) on Ca absorption and peanut yields. They reported the greatest effects of sorbitol-chelated Ca application on improving foliar nutrient uptake and crop yield. However, the negatively charged property of metal chelates should be considered before use, because it influences the processes of diffusion and translocation of nutrients (Fernández and Brown, 2013). The efficacy of chelated fertilizers is modulated by their properties (e.g., physicochemical, structural, chelating-strength aspects, Niu et al., 2021) and leaf epidermal traits (e.g., cuticles, wax layers, stomata, trichomes or lenticels). The study by Li et al. revealed a high use efficiency of sorbitol-chelated Ca due to sorbitol-mediated wetting effect on contact angle. In addition, foliar spraying of the chelated agency stimulates Ca accumulation of subcellular fractions (e.g., cell wall and organelles) and re-orients Ca distribution in mesophyll tissue; these alterations improve the crop quality and yield.

Nanoparticles are increasingly used as innovative nutrient delivery system due to the advantages such as high surface area to volume ratio, superior sorption capacity, and controlled-release (Solanki et al., 2015). Increasing evidence has demonstrated the benefit of foliar-applied nano macronutrients (e.g., nano N, P, K) and micro-nutrients (e.g., nano Fe, Cu, Zn and Mn) on soil health and crop production and quality (Al-Juthery et al., 2021). For example, foliar application of silicon nanoparticles (nSiO_2_) enhanced safflower production (Anmohammadi et al., 2016). The effects of foliar-applied nanofertilizers involve seed germination, physiology (such as photosynthetic activity) and metabolism (such as carbohydrate and protein synthesis, plant growth, and yield (Solanki et al., 2015). Nanoparticles enter leaf mainly through stomata, with a little of cuticle permeability, and then are metabolized locally or transported to other plant tissues *via* apoplastic and symplastic pathways Fernández et al., 2013; Solanki et al., 2015). The species and environmental factors and physicochemical properties of nanoparticles co-determine foliage uptake and nutrient use efficiency of nano fertilizers (Hong et al., 2021). The development and the application of nano-fertilizers have enormous potential to increase agricultural productivity (Al-Juthery et al., 2021). However, the increasing release of nano-materials into the ecosystems and the food chains may raise the potential risk to human health and ecological safety (Zulfiqar et al., 2019). Therefore, a comprehensive evaluation system should be constructed and improved biotechnologies are required to use for foliar application of nano-fertilizers in agriculture (Zulfiqar et al., 2019).

The structure and the chemistry of leaf surface are important in determining the bi-directional diffusion of substances between leaf surface and surrounding environment and the use efficiency of foliar-applied nutrients (Fernández et al., 2013). Micro-scopic amounts and/or large volumes of water visible commonly exist on leaf surfaces (Tredenick et al.). Macro-scopic and/or micro-scopic leaf wetness have the significance in the foliar exchange of ions and atmospheric trace gases (Burkhardt and Hunsche, 2013). Moreover, leaf wetness usually influences phyllospheric organisms (e.g., fungi, bacteria or insects), further phyllospheric microganism-mediated leaf structure and physiology (Burkhardt and Hunsche, 2013). In this Research Topic, Tredenick et al. identified the materials on the leaf surface and quantified the hygroscopicity of these materials. The materials on leaf surface are mixtures of hygroscopic salts (e.g., NaCl and KCl), minerals (e.g., Al, Mn, and Zn), lipophilic compounds (e.g., oils and waxes), and others. These materials are hygroscopic, thus help to form an aqueous solution in the micro-environments. In addition, the hygroscopic materials can alter surface tension of droplets, solubleness of the applied ion matters (Fernández et al., 2017), the other properties (e.g., evaporation time, contact angle and area of droplets), and total amount of penetrated chemicals (Tredenick et al.). Leaf surface materials can increase hydraulic activation of stomata, further influence foliar water and nutrient fluxes through the liquid water networks between the leaf surface and the apoplast along the stomatal walls (Burkhardt and Hunsche, 2013). Therefore, the properties of leaf surface materials directly influence the processes such as penetration of foliar-applied nutrients, foliar wetness and water uptake, and stomatal apertures and distribution (Tredenick et al.). In a canopy scale, leaf surface materials are potential regulators in the trade-off between hydraulic efficiency and safety (Burkhardt and Hunsche, 2013).

Plant species are significantly divergent in the resistance and absorption capacity when they are exposed to atmospheric aerosols. Singh et al. revealed the species ramifications of leaf surface materials on nutrient allocation patterns and the rates of translocation to plant tissues. They found significant differences in tissue levels of macro- (such as C, N, and K) and micro-nutrients (such as Zn, Mn) and translocation rates between evergreen and deciduous species. They concluded that the deciduous species (such as *Dalbergia sissoo*) were tolerant to foliar deposition of particulate matters and the semi-evergreen tree species (*Ficus religiosa* and *Azadirachta indica*) had high re-translocation efficiency. Wu et al. found the physiological roles of canopy N addition in improvement in the photosynthetic rate and revealed a potential mechanism in view of hydraulic conductivity and distance of CO_2_ transportation in leaves. Uptake of atmospheric aerosols involves complex but poorly understood metabolic networks and regulatory processes. The atmospheric aerosols are commonly hygroscopic in the humidity conditions (e.g., leaf surfaces close to transpiring stomata, Chi et al.). This physiochemical property is beneficial to foliar uptake of the applied nutrient solution. Chi et al. studied the potential impacts of atmospheric aerosols on physiochemical properties and physiological variables of *Cinnamomum camphora*; and found that the plants grown in an air-filtered condition developed the leaves with less amorphous, flat areas but this type of leaves were abundant for plants exposed to the ambient air. On the basis, Chi et al. concluded that the amount and the type of leaf surface materials, atmospheric conditions, and plant ecophysiological traits co-determinated plant water relations. They further proposed to use amorphous areas as a potential indicator representing the deposition level of atmospheric aerosols.

In the past decades, foliar fertilization has been widely used as a supplementary measure to soil fertilizer application for improvement in crop quality and yield. However, a series of ecological and safety issues raised in accompanied with foliar fertilization practice (Oosterhuis, 2009). Obviously, larger variations in plants exist in the efficiency of nutrient delivery system, their translocation and distribution in plant tissues, and growth and yield performances. These are probably associated with foliar application regimes (e.g., timing of applications, type of fertilizers, and soil nutrient availability), species traits, and environmental factors. In addition, the uptake and the use efficiency of foliar-applied nutrients depend on the rates of translocation to different plant tissues (Fernández et al., 2013). Use efficiency of foliar-applied matters is a core principle for assessing its utilization in a crop production system (Al-Juthery et al., 2021). However, many aspects related to the uptake and mobility of foliar-applied nutrients are still not known (Fernández et al., 2013). It is well known the apoplastic movement of foliar-applied nutrients (Fernández and Brown, 2013); while, to date, it is still not well known what and how the factors (e.g., cell wall charge, pore size, pH, and ionic strength) within the apoplast regulate the movement and the interaction of foliar-applied nutrients in and through the apoplastic space. Excess application of foliar fertilizers can result in nutrient toxicity then limit crop growth (Al-Juthery et al., 2021). Moreover, soil and foliar application of fertilizers can disturb plant N_2_O emission. The study by Zhu et al. revealed that long-term nitrogen addition profoundly stimulated cumulative plant N_2_O emissions, which is an important factor disturbing the atmosphere’s N_2_O budget in the ecosystems. The results of Liu et al. indicated that short-termly simulated N deposition enhanced the drought tolerance of evergreen broad-leaved trees by osmotic adjustment and protecting the photosystem. However, long-term N deposition may result in increasing plant water loss or deficiency, even forest degradation. Carnivorous plants are the important components of terrestrial and aquatic ecosystems, with great capable of foliar uptake of mineral nutrients (e.g., N and P). However, the factors affecting the uptake, translocation, and use efficiency of foliar-applied nutrients by carnivorous plants are still unknown (Adamec, 2013).

## Author contributions

YH wrote the Research Topic proposal and Editorial. YH organized as a leading editor the Research Topic; NB and YK co-organized the Research Topic and revised the Editorial. All authors contributed to the article and approved the submitted version.

## References

[B1] Adamec L. (2013). Foliar mineral nutrient uptake in carnivorous plants: what do we know and what should we know? Front. Plant Sci. 4. doi: 10.3389/fpls.2013.00010 PMC3560283 23386858

[B2] Alexander A. Schroeder M. (1987). Fertilizer use efficiency: Modern trends in foliar fertilization. J. Plant Nutr. 10, 9–16. doi: 10.1080/01904168709363671

[B3] Al-Juthery H. W. A. Lahmod N. R. Al-Taee R. A. H. G. (2021). “Intelligent, nano-fertilizers: A new technology for improvement nutrient use efficiency,” in IOP Conference Series: Earth and Environmental Science, Vol. 735. 012086. doi: 10.1088/1755-1315/735/1/012086

[B4] Anmohammadi M. Amanzadeh T. Sabaghnia N. Ion V. (2016). Effect of nano-silicon foliar application on safflower growth under organic and inorganic fertilizer regimes. Bot. Lith 22 (1), 53–64. doi: 10.1515/botlit-2016-0005

[B5] Azam M. Bhatti H. N. Khan A. Zafar L. Iqbal M. (2022). Zinc oxide nano-fertilizer application (foliar and soil) effect on the growth, photosynthetic pigments and antioxidant system of maize cultivar. Biocatal. Agr Biotechnol. 42, 102343. doi: 10.1016/j.bcab.2022.102343

[B6] Bellaloui N. Hu Y. B. Mengistu A. Kassem M. A. Abel C. A. (2013). Effects of foliar boron application on seed composition, cell wall boron, and seed δ^15^N and δ^13^C isotopes in water-stressed soybean plants. Front. Plant Sci. 4. doi: 10.3389/fpls.2013.00270 PMC3719013 23888163

[B7] Burkhardt J. Grantz D. A. (2016). “Plants and atmospheric aerosols,” in Progress in botany, vol. Vol. 78 . Eds. Cánovas F. Lüttge U. Matyssek R. (Cham: Springer). doi: 10.1007/124_2016_12

[B8] Burkhardt J. Hunsche M. (2013). “Breath figures” on leaf surfaces–formation and effects of microscopic leaf wetness. Front. Plant Sci. 4. doi: 10.3389/fpls.2013.00422 PMC3807045 24167510

[B9] Fernández V. Bahamonde H. A. Peguero-Pina J. J. Gil-Pelegrín E. Sancho-Knapik D. Gil L.. (2017). Physico-chemical properties of plant cuticles and their functional and ecological significance. J. Exp. Bot. 68, 5293–5306. doi: 10.1093/jxb/erx302 28992247

[B10] Fernández V. Brown P. H. (2013). From plant surface to plant metabolism: the uncertain fate of foliar-applied nutrients. Front. Plant Sci. 4. doi: 10.3389/fpls.2013.00289 PMC3728483 23914198

[B11] Fernández V. Sotiropoulos T. Brown P. H. (2013). Foliar fertilization: Scientific principles and field pratices. 1st ed. (Paris, France: International Fertilizer Industry Association (IFA).

[B12] Hong J. Wang C. Wagner D. C. Gardea-Torresdey J. L. He F. Rico C. M. (2021). Foliar application of nanoparticles: mechanisms of absorption, transfer, and multiple impacts. Environ. Sci.: Nano 8, 1196–1210. doi: 10.1039/D0EN01129K

[B13] Hu Y. B. Fernández V. Ma L. (2014). Nitrate transporters in leaves and their potential roles in foliar uptake of nitrogen dioxide. Front. Plant Sci. 5. doi: 10.3389/fpls.2014.00360 PMC4115617 25126090

[B14] Mengel K. (2002). “Alternative or complementary role of foliar supply in mineral nutrition,” in ISHS acta horticulturae 594 (International Symposium on Foliar Nutrition of Perennial Fruit Plants). 594, 33–47.

[B15] Niu J. H. Liu C. Huang M. L. Liu K. Z. Yan D. Y. (2021). Effects of foliar fertilization: a review of current status and future perspectives. J. Soil Sci. Plant Nutr. 21, 104–118. doi: 10.1007/s42729-020-00346-3

[B16] Oosterhuis D. (2009). Foliar fertilization: Mechanisms and magnitude of nutrient uptake. Pap. Fluid Fertilizer Foundat0 meeting Scottsdale Arizona. February 15-17, 2009.

[B17] Solanki P. Bhargava A. Chhipa H. Jain N. Panwar J. (2015). “Nano-fertilizers and their smart delivery system,” in Nanotechnologies in food and agriculture. Eds. Rai M. Ribeiro C. Mattoso L. Duran N. (Cham: Springer). doi: 10.1007/978-3-319-14024-7_4

[B18] Souri M. K. Hatamian M. (2019). Aminochelates in plant nutrition: a review. J. Plant Nutr. 42 (1), 67–78. doi: 10.1080/01904167.2018.1549671

[B19] Zheng X. C. Wang H. B. Wang X. D. Wang B. L. Shi X. B. Liu F. Z. (2016). Researches on selenium absorption and distribution in kyoho grape. Chin. Soils Fert (4), 128–132. doi: 10.11838/sfsc.20160422

[B20] Zulfiqar F. Navarro M. Ashraf M. Akram N. A. Munné-Bosch S. (2019). Nanofertilizer use for sustainable agriculture: Advantages and limitations. Plant Sci. 289, 110270. doi: 10.1016/j.plantsci.2019.110270 31623775

